# *Wolbachia w*Mel and *w*AlbB strains differentially impact the vector competence of *Aedes aegypti* with a Brazilian genetic background for DENV-1 virus

**DOI:** 10.1186/s13071-026-07292-6

**Published:** 2026-03-05

**Authors:** Carolina Boucinha Martins, Mariana Rocha David, Dinair Couto-Lima, Jessica Corrêa-Antônio, Rayane Teles-de-Freitas, Manuella Mello-Barbosa, Renke Lühken, Ary Hoffmann, Rafael Maciel-de-Freitas, Márcio Galvão Pavan

**Affiliations:** 1 Laboratório de Mosquitos Transmissores de Hematozoários, Instituto Oswaldo Cruz, Fiocruz, Rio de Janeiro, Brazil; 2 Department of Entomology and Arbovirology, Bernhard Nocht Institute for Tropical Medicine, Hamburg, Germany; 3 Research Group Vector Control, Bernhard Nocht Institute for Tropical Medicine, Hamburg, Germany; 4 Pest and Environmental Adaptation Research Group, School of BioSciences, Bio21 Institute, The University of Melbourne, Melbourne, Australia

**Keywords:** *Wolbachia*, *Aedes aegypti*, Dengue, Viral blocking, Brazil

## Abstract

**Background:**

*Aedes aegypti* mosquitoes infected with the endosymbiotic bacterium *Wolbachia pipientis* have been released as a sustainable strategy to mitigate arbovirus transmission. Among the strains successfully deployed, *w*Mel and *w*AlbB have shown promising blocking effects against dengue virus (DENV). However, the strength of viral inhibition depends on *Wolbachia* density within mosquito tissues, the genetic backgrounds of both host and virus, and the viral dose. In this study, we aimed to investigate the vector competence for DENV-1 of *Ae. aegypti* with Brazilian genetic background infected with *w*Mel or *w*AlbB.

**Methods:**

A total of 493 wild and *w*Mel- and *w*AlbB-infected mosquitoes were orally challenged with low (5 × 10^4^ FFU/mL) and high (5 × 10^5^) titers of DENV-1. Relative *Wolbachia* density was measured by quantitative polymerase chain reaction (qPCR), and viral infection in mosquito bodies and saliva was assessed by qPCR with reverse transcription (RT-qPCR). Transmission potential was tested through saliva microinjection into susceptible mosquitoes. The infection prevalence and viral loads in mosquito bodies were analyzed at 7, 14, and 21 days post infection (dpi).

**Results:**

Both *Ae. aegypti* groups infected with *w*Mel and *w*AlbB had reduced (albeit distinct) DENV-1 infection and transmission relative to wild type mosquitoes. *w*Mel-infected mosquitoes exhibited less abundant bacteria in their bodies but a greater degree of DENV-1 inhibition compared with those carrying *w*AlbB, indicating that DENV-1 blocking is strain specific rather than *Wolbachia* density-driven. Moreover, we observed that *Wolbachia* had a protective effect on mosquitoes by decreasing the DENV-1 loads in their bodies, but with a constant presence of virus. Viral transmission rates in the saliva were similar among wild and *w*Mel- and *w*AlbB-infected mosquitoes at 7 and 14 dpi but lower in *w*Mel and *w*AlbB mosquitoes at 21 dpi.

**Conclusions:**

The similar DENV-1 loads in mosquito bodies over time (7, 14, and 21 dpi) infected with either the *w*Mel or *w*AlbB strain, regardless of the viral titer of the infectious blood meal, suggest that *Wolbachia* may have a maximum pathogen-blocking capacity beyond which additional virus suppression cannot be achieved. The viral suppression only after 21 dpi in the saliva raises concerns and warrants further investigation, as females may transmit before *Wolbachia* blockage becomes effective. While *w*AlbB may exhibit comparable DENV-1 blocking to *w*Mel, its enhanced thermal tolerance makes it epidemiologically relevant in tropical regions. Continuous monitoring of *Wolbachia* dynamics and DENV genomic variation in the field remains essential to evaluate long-term effectiveness and detect potential adaptive viral responses.

**Graphical Abstract:**

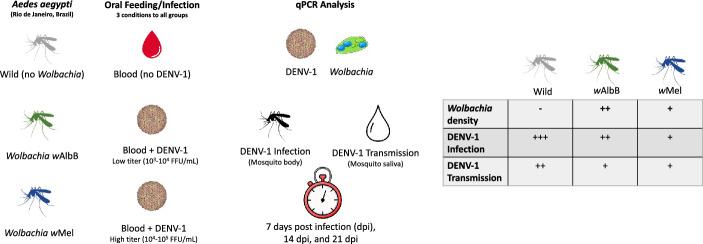

**Supplementary Information:**

The online version contains supplementary material available at 10.1186/s13071-026-07292-6.

## Background

The incidence of dengue (DENV) has increased tenfold in the past 20 years, reaching 7.6 million reported cases, accounting for over 80% of arthropod-borne virus (arbovirus) cases in the Americas [1]. The *Aedes aegypti* (Diptera: Culicidae) mosquito is the main dengue vector largely due to its close association with humans, as females preferentially feed on human blood and lay eggs in artificial containers near human habitations [2]. The recurrence of dengue outbreaks across the Americas demonstrates that traditional vector control approaches, including mechanical and chemical strategies, have been insufficient to achieve sustainable suppression of mosquito populations [3–6]. Therefore, there is an urgent need for implementing new methods to reduce the incidence of arboviruses.

An innovative approach to mitigate dengue transmission is the deployment of *Ae. aegypti* mosquitoes infected with the endosymbiotic bacterium *Wolbachia pipientis* (Rickettsiales, Anaplasmataceae) in endemic areas [7]. *Wolbachia* is naturally present in many insects, but not in *Ae. aegypti*. Overall, it is maternally inherited with high fidelity in both laboratory colonies and under field conditions [8, 9], and its maintenance in field mosquitoes is facilitated by a phenomenon known as cytoplasmic incompatibility, in which chromosomal misalignments occur and which affects the viability of embryos when females without *Wolbachia* are inseminated by males with the bacterium [10, 11]. Moreover, it has been shown that *Wolbachia* interferes with the mosquito’s ability to transmit different arboviruses, including DENV, Zika virus, and chikungunya virus [7, 12–15].

Different *Wolbachia* strains have been transinfected into *Ae. aegypti* [7, 14, 16]. Among them, the *w*Mel strain, from the fly *Drosophila melanogaster* (Diptera: Drosophilidae), and the *w*AlbB strain, from *Aedes albopictus*, have been the most widely explored so far in field trials. *Wolbachia* can be used under two different strategies: population suppression, by releasing male mosquitoes with *Wolbachia*, or replacement, releasing both male and female mosquitoes aiming to substitute wild mosquito populations highly susceptible to arboviruses with *Wolbachia*-infected mosquitoes less competent to arbovirus infections [17–19]. For instance, a successful population replacement was achieved in sites in Malaysia when releasing *Ae. aegypti* mosquitoes with the *w*AlbB strain (reaching 82.3%), leading to an average reduction in dengue incidence of 62% [20]. *Aedes aegypti* infected with the *w*Mel strain has been deployed since 2011, starting in Australia, where dengue incidence reduced > 95% with this strategy [8, 21]. Significant reductions in dengue incidence of 77% and 96% were also observed in Indonesia and Colombia, respectively, demonstrating the effectiveness of the method [22, 23]. Inundative releases of *Ae. aegypti* infected with *w*Mel also occurred from 2017 to 2019 in Rio de Janeiro, Brazil, but achieved more modest results, with a reduction of 38% in dengue cases [24].

Despite *w*Mel and *w*AlbB seeming to have similar arbovirus blocking effects [18], the strength of *Wolbachia*-mediated viral inhibition may depend on the bacterium density in mosquito tissues [25], which can be affected by both the host and viral genetic backgrounds [26]. The blocking phenotype of *Wolbachia* also appears to be affected by the viral titer. For instance, *w*Mel–*Ae. aegypti* with a Brazilian genetic background orally challenged with a low DENV-1 titer (3 × 10^4^ FFU/mL) were less susceptible to viral infection than wild mosquitoes. However, when exposed to a high DENV-1 titer (6 × 10^8^ FFU/mL), *w*Mel mosquitoes displayed DENV-1 infection rates comparable to those of wild counterparts, although their bodies and saliva exhibited lower viral loads [27]. Considering that *Wolbachia* is an alternative approach to mitigate dengue transmission, it is of epidemiological relevance to test the arbovirus blocking effect of different *Wolbachia* strains in *Ae. aegypti* mosquitoes. Here, this work aimed to explore the vector competence for DENV-1 of *w*Mel- and *w*AlbB-infected *Ae. aegypti* mosquitoes with a Brazilian genetic background.

## Methods

### Mosquitoes used in experimental oral infections

Wild (i.e., *Wolbachia*-uninfected) *Ae*. *aegypti* mosquitoes were sampled in Urca (22° 57′ 15′′ S; 43° 10′ 3′′ W), a neighborhood of the city of Rio de Janeiro, Brazil, with 60 ovitraps evenly distributed to obtain a minimum of 5000 eggs to represent the genetic diversity of the mosquito population. Mosquitoes with the *Wolbachia w*AlbB strain were derived from Australia [28] and those with the *w*Mel strain were obtained from the field, in Tubiacanga (22˚47′06″ S; 43˚13′32″ W)—an isolated neighborhood in Rio where *Wolbachia* releases started in 2015 and, since then, the *w*Mel frequency has been near 100% [29]. Females infected with the two *Wolbachia* strains were backcrossed with F1 male mosquitoes from Urca for five consecutive generations in the laboratory to produce *Wolbachia*-infected lines with the same Brazilian genetic background. Subsequently, every two generations, the laboratory-reared female mosquitoes were outcrossed to F1 males from Urca at a 1:2 ratio of males and females per cage (around 600:1200 per cage) to ensure genetic diversity in the colonies and to ensure that the material used in the experiments had a similar genetic background to those of field mosquitoes, allowing phenotypic effects specifically associated with *Wolbachia* [30, 31] to be isolated. These colonies were tested through quantitative polymerase chain reaction (qPCR) every generation to confirm *Wolbachia* infection that was consistently > 95% (data not shown).

### *Wolbachia* quantification in *Ae. aegypti*

DNA was extracted individually from each sample using the NucleoSpin^®^ Tissue kit (Macherey–Nagel, Düren, Germany), according to the manufacturer’s protocol. Relative *Wolbachia* density was quantified by qPCR using SYBR Green-based real-time PCR with primers previously designed by our group to amplify a 115-base pair (bp) fragment of the *Wolbachia* surface protein gene (*wsp*; GenBank gene ID no. CP090948) in *w*Mel and a 106-bp fragment of the *Ae. aegypti* ribosomal protein S6 gene (*rps6*; GenBank gene ID no. 5563590) [32] (Table 1). In addition, we designed a pair of primers that amplifies 123 bp of *wsp* in *w*AlbB, using the same strategy outlined elsewhere [32]. In brief, genomic sequences were retrieved from the Bacterial and Viral Bioinformatics Resource Center v.3.49 (https://www.bv-brc.org/) and from the National Center for Biotechnology Information (NCBI) (https://ncbi.nlm.nih.gov/) and subsequently aligned using the MultAlin platform (http://multalin.toulouse.inra.fr/multalin/). For the *wsp* gene, sequences from different *Wolbachia* strains (*w*Mel, *w*AlbA, and *w*AlbB) were compared to identify a region suitable for specific amplification of the *w*AlbB and *w*Mel strains. In silico primer design was carried out using Primer3 (https://primer3.ut.ee/), targeting fragments between 50 and 150 bp. Primer quality was further assessed with an online evaluation tool (https://www.idtdna.com), considering thermodynamic parameters relevant for qPCR performance. Primers were selected with melting temperatures (Tm) > 45 °C, a maximum difference of 5 °C between forward and reverse primers, and Gibbs free energy (ΔG) values above −3 kcal/mol for hairpin formation and above −10 kcal/mol for dimer formation. While the forward primer designed to amplify a fragment of the *w*Mel *wsp* gene is highly conserved among *Wolbachia w*Mel, *w*AlbA, and *w*AlbB strains (sharing all 27 nucleotides with *w*AlbA and differing in only three nucleotides from *w*AlbB), the reverse primer has greater specificity for *w*Mel, containing ten nucleotides unique to this strain and eight nucleotides shared between *w*Mel and *w*AlbA. However, while the reverse primer designed to amplify a fragment of the *w*AlbB *wsp* gene shares all nucleotides with *w*AlbA and differs from *w*Mel in only 1 out of 20 nucleotides, the forward primer has 4 and 5 nucleotides that differ from those of the *w*AlbA and *w*Mel strains, respectively (Table 1).

**Table 1:**
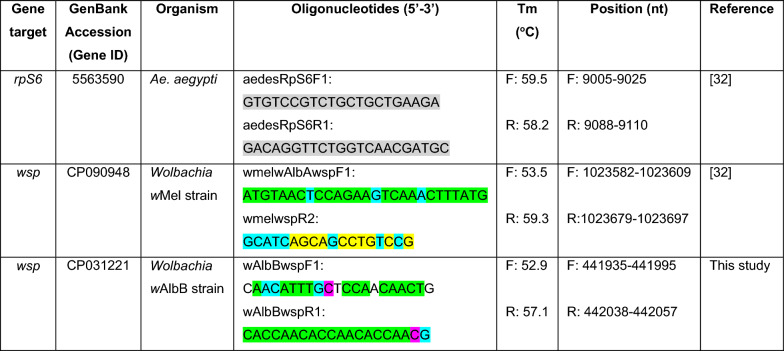
Oligonucleotides used in this study

Details of the oligonucleotides are provided, including their gene and organism targets, accession IDs, genomic positions, their sequences, and where they were published. Color key: grey, gene exon; green, regions conserved among *w*Mel, *w*AlbA, and *w*AlbB; cyan, regions shared between *w*Mel and *w*AlbA; yellow, regions unique to *w*Mel; white, regions unique to *w*AlbB; pink, regions shared between *w*AlbA and *w*AlbB

*Aedes aegypti* mosquitoes were used to evaluate primer efficiency and specificity, including *w*Mel-infected individuals (F5 generation from Tubiacanga, Rio de Janeiro, Brazil) and *Wolbachia*-free mosquitoes (Paea strain, derived from mosquitoes caught in French Polynesia in 1994 and maintained for more than 400 generations). Mosquitoes were reared and maintained in an incubator (Solab SL-200/364) under controlled conditions (28 °C, 12 h light:12 h dark). Female adults aged 0–3 days were flash-frozen in liquid nitrogen and were homogenized individually with pestles. Homogenates were preserved in 350 µL Buffer RLT Plus (AllPrep DNA/RNA Mini Kit, Qiagen, Hilden, Germany) at −80 °C until nucleic acid extraction. Total DNA and RNA were extracted with the AllPrep DNA/RNA Mini Kit (Qiagen, Hilden, Germany), following the manufacturer’s instructions, and DNA was used for primer validations. DNA concentration was measured with a Qubit 4 Fluorometer (Thermo Fisher Scientific, Waltham, USA). Pools of ten individuals (~10 ng/µL DNA per sample) were prepared for both *w*Mel-positive and *Wolbachia*-free controls, and serial dilutions (1/1 to 1/32; 12.5–400 ng total DNA) were performed to determine primer efficiency and specificity. qPCR reactions were run on a QuantStudio™ 6 Flex Real-Time PCR System (Applied Biosystems, Waltham, MA, USA) using a 15 µL mix containing 7.5 µL Power SYBR Green PCR Master Mix (Thermo Fisher Scientific), 0.75 µL of each primer (10 mM), 4 µL DNA template, and 2 µL water. Cycling conditions were: 50 °C for 2 min, 95 °C for 10 min, followed by 40 cycles of 95 °C for 15 s and 60 °C for 1 min. Melt curve analysis was carried out at 95 °C for 15 s, 60 °C for 1 min, and then a gradual increase back to 95 °C for 15 s. Each assay was performed as three independent replicates.

Relative quantification was done through computing the delta threshold cycle (ΔCt), obtained from the difference between the Ct values from the *Wolbachia wsp* gene and the *rps6* control gene from the mosquito. The results were then normalized with the lowest delta Ct value obtained from all samples analyzed in this study. Positive (*Ae. aegypti* infected with *w*Mel or *w*AlbB) and negative controls (reagents + water, mosquito without *Wolbachia*) were included in all qPCR assays to ensure the reliability of the results.

### Dengue-1 virus used in oral experimental infections

The dengue-1 virus MV09 strain used in the experimental infection assays was isolated from a human case in 2015 in the state of Minas Gerais, Brazil (DENV1/*Homo sapiens*/Brasil/Contagem/MG/MV09/2015), and it was in the second passage. The virus was cultivated in C6/36 cells (48th passage) in Leibowitz L-15 medium supplemented with 10% fetal bovine serum (FBS), 1% penicillin/streptomycin (Gibco, Thermo Fisher Scientific, CA, USA), and 2% nonessential amino acids without l-glutamine (Sigma-Aldrich, St. Louis, USA). Fresh supernatant from infected C6/36 cells was harvested 5 days after infection and stored in 1 mL aliquots at −80 °C until use. For viral titration, the viral envelope protein E was detected by immunofluorescence in C6/36 cultures using DENV-specific monoclonal antibodies (purified from ascitic fluid anti-dengue virus 1; in-house lab LATAM production; product batch: 041118FDEN1P; technical expert: Tiago Pereira). The focus-forming units (FFUs) were counted in an EVOS^®^ FL Auto Imaging System (Life Technologies, Carlsbad, CA, USA). The viral stock had an initial titer of 5 × 10^5^ FFU/mL when mosquitoes began feeding on the infected blood and showed an approximate 1-log reduction by the end of the experiments (5.4–6.2 × 10^4^ FFU/mL). All experiments were performed using this same viral stock at the original titer and at a tenfold dilution (5 × 10^4^ FFU/mL). A similar reduction was observed in the diluted stock during the experiments (4.8–5.7 × 10^3^ FFU/mL). Therefore, we refer to conditions in which mosquitoes fed on 10^4^–10^5^ FFU/mL of DENV-1 as “high titer” and on 10^3^–10^4^ FFU/mL as “low titer.”

### Oral feeding of mosquitoes with DENV-1

Oral infection assays were performed with females of each group (*Wolbachia*-uninfected *Ae. aegypti*, hereafter called “wild,” *w*Mel-infected, or *w*AlbB-infected), maintained at 28 ± 1 °C in cages with sugar solution ad libitum (10%) until they were 5–6 days old. One day before viral exposure, female mosquitoes were deprived of sugar solution overnight and in the next morning were transferred to cylindrical cages (8 cm height, 6 cm diameter, with 35 females per cage). The DENV-1 strain used herein efficiently infects mosquitoes by thawing the virus and offering it to the mosquitoes with blood. Female mosquitoes of each group were thus exposed to two (low and high) DENV-1 titers by oral feeding in an artificial feeder (Hemotek, Great Hardwood, UK) at 37 °C for approximately 30 min. Infected blood meals contained 1 mL of erythrocytes with human blood (approved by Fiocruz Ethics Committee–CAAE 53419815.9.0000.5248), and 1 mL of virus suspended in L15 culture medium (Leibovitz). The remaining 200 female mosquitoes per group were fed on 1 mL of erythrocytes and 1 mL of L15 medium free of virus. At least one cage from each experimental group and condition was placed simultaneously on the artificial feeders to minimize variation in the amount of virus ingested during the blood meal. Only females fully engorged were selected for further analysis, being maintained in Biochemical Oxygen Demand incubators with controlled temperature (28 ± 1 °C), humidity (60%), and light and dark conditions (12:12 h) in 50 mL plastic cages containing no more than 20 females per cage. Non-visually fed mosquitoes were discarded. All experimental conditions were conducted in two biological replicates.

### Salivation and intrathoracic saliva microinjection

At 7, 14, and 21 days after feeding on blood (exposed or unexposed to DENV-1), mosquito saliva was collected from 30 females of each group. Females were briefly anesthetized on ice, their wings and legs removed, and the proboscis inserted into sterile filtered 10µL pipette tips containing 10µL of sterile fetal bovine serum (Gibco) with blue food coloring for 30 min. Only females with blueish abdomens were kept in a –80 °C freezer for further molecular analysis.

The saliva from up to 15 mosquitoes per group that had DENV-1 infection confirmed by qPCR with reverse transcription (RT-qPCR) in their bodies was collected and tested individually. Each saliva sample was intrathoracically injected (69 nL) with pulled-glass capillary needles into 15 DENV-1-susceptible *Ae. aegypti* females from Urca, Rio de Janeiro, Brazil, using a NanoJect II microinjector (Drummond Scientific Company), as described by Moreira et al. [12]. Mosquitoes were anesthetized on ice for 5 min, and the needle was inserted in the thoracic intersegmental membrane. This step was performed by a single trained operator to minimize human bias in the results. The saliva of females not exposed to DENV-1 was microinjected as a negative control. The microinjected females were kept in a Biochemical Oxygen Demand (BOD) incubator with the same condition as described above. The microinjected individuals were collected 7 days after microinjection and frozen at –80 °C for future analysis and detection of viral RNA. Mortality rates of microinjected mosquitoes were checked 24 h and 7 days after injections (end of the experiment) and, when above 30%, the procedure was repeated. The mortality rates between *Ae. aegypti* infected with *Wolbachia* were low (overall < 20%) and similar to those observed for the control. The results were interpreted such that if at least one injected female tested positive for DENV-1, the corresponding mosquito that provided the saliva sample was considered positive for transmission.

### Confirmation of DENV-1 infection and transmission in orally fed *Ae. aegypti*

The extraction of viral RNA from the whole body of orally fed or intrathoracically injected mosquitoes was performed with a QlAamp Viral RNA Mini kit (Qiagen, Hilden, Germany) according to the manufacturer’s instructions. The DENV-1 viral load was quantified by the number of RNA copies present in the entire body of each mosquito through a one-step Master Mix (Applied Biosystems, Waltham, MA, USA) using the QuantStudio 6 Flex Real-Time PCR System (Applied Biosystems, Waltham, MA, USA). We used the TaqMan probe (Thermo Fisher Scientific, CA, USA) to detect DENV-1 in the samples. The blank and negative controls were composed of the qPCR reaction mix + water and the qPCR reaction mix + RNA from mosquitoes fed only with DENV-1-free blood. Each reaction was made with previously published primers (DENV-1 F: 5′ CAAAAGGAAGTCGTGCAATA 3′, DENV-1 C: 5′ CTGAGTGAATTCTCTCTACTGAACC 3′) and probe (DENV-1 probe: 5′ CATGTGGTTGGGAGCACGC 3′ FAM/BHQ-1) [33]. Amplification conditions consisted of 12 nmol of forward and reverse primers, 9 nmol of probe, 5µL of TaqMan™ Fast Virus 1-Step Master Mix (Applied Biosystems), and 5µL of RNA. Cycling conditions were as follows: 45 °C for 15 min, 95 °C for 20 s, followed by 40 amplification cycles of 95 °C for 15 s, 58 °C for 5 s and 60 °C for 30 s. Absolute viral quantification was performed through interpolation onto an internal standard curve composed of a six-point dilutions series (10^1^–10^6^ DENV-1 copies).

Limit of detection (LOD) analyses were done on the basis of eight replicates of a 10-point dilution series (10^6^–10^−3^ copies). Calculations were performed in the R environment (version 3.6.2) using probit regression with binary data (1 = detected through qPCR; 0 = not detected) as the dependent variable and the log_10_-transformed input concentration as the independent variable. Probit models were fitted using the glm function with a probit link. The fitted regression parameters were used to estimate the concentration corresponding to 95% probability of detection (LOD95). The 95% confidence interval (CI) for the LOD95 estimate was then obtained using nonparametric bootstrap resampling, with 1000 replicates. In our case, the LOD95 = 1.75 viral copies (95% CI 0.42–2.37 viral copies).

### Statistical analysis

#### Relationship between relative *Wolbachia* density and days post DENV-1 infection and viral titer in orally infected mosquitoes

A generalized linear model (GLM) with a gamma distribution and a log link function were used owing to the positive and skewed nature of the response variable (relative *Wolbachia* density), for which we tested the association with dpi and viral titer (explanatory variables) for *w*Mel and *w*AlbB strains. Only mosquitoes infected with *Wolbachia* were considered and the full models included the main effects and the two-way interaction between explanatory variables. To simplify the models while retaining explanatory power, we compared the models with and without the interaction term using likelihood ratio tests. If the interaction did not significantly improve model fit, the simpler model was retained for interpretation. Model fit was examined by checking heteroscedasticity, residual dispersion, and the presence of outliers with the R package DHARMa [34]. The relative *Wolbachia* density was also compared over time post infection for each group and between groups (*w*Mel and *w*AlbB) at each dpi using Mann–Whitney tests (*W*) and *P*-values adjusted using the Benjamini–Hochberg false discovery rate (BH-FDR) procedure [35]. All analyses were performed in the R environment (version 3.6.2).

#### Association of DENV-1 body infection and transmission with mosquito group (wild, *w*Mel, and *w*AlbB), days post infection and viral titer in orally infected mosquitoes

DENV-1 infection rates of the mosquito’s body and saliva (*i.e.* transmission rate, qualitative analysis), as well as the number of viral copies detected in DENV-positive *Ae. aegypti* (quantitative analysis) were analyzed. Logistic regressions with infection status (“yes” or “no”) were used as the dependent variable to calculate DENV-1 infection/transmission rates. The models were fitted following two complementary approaches: (i) for each dpi (7, 14, and 21), we assessed the effect of *Wolbachia* by comparing mosquito groups (wild, *w*Mel, and *w*AlbB) and viral titers (low and high) as explanatory variables; and (ii) within each mosquito group (wild, *w*Mel–, and *w*AlbB–*Ae. aegypti*), we investigated infection and transmission dynamics across time points (7, 14, and 21 dpi) and viral titers (low and high) as explanatory variables. Models with and without interactions were compared via likelihood ratio tests, and model fit was checked using the estimates and standard errors via the DHARMa R package [34]. In the cases when the likelihood ratio test supported the addition of an interaction term, but convergence issues were observed (e.g., quasi-complete separation, inflated standard errors, or unreliable coefficient estimates), we opted to report the simpler additive model. Model results were expressed as odd ratios, with 95% confidence intervals and *P*-values. In addition, proportions of DENV infection (yes/no) between different experimental conditions (*e.g.,* group or dpi under the same viral titer) were compared using contingency chi-squared or Fisher’s exact tests (the latter used when any expected cell count was < 5). *P*-values were adjusted using the BH-FDR procedure to control for multiple testing.

For quantitative analysis, the viral load data in orally infected *Ae. aegypti*, which exhibited right skewness, was log_10_ transformed and analyzed through linear models fitted per (i) dpi (with group and viral titer as explanatory variables) and (ii) group of mosquitoes (with dpi and viral titer as independent variables). Models with and without interactions were compared via *F*-tests, and model fit was checked following the above methods. Results were expressed as estimates, with 95% confidence intervals and *P*-values. DENV infection load was also compared between groups and time points under the same viral titer through Mann–Whitney tests, adjusting the *P*-values with BH-FDR for multiple comparisons. Linear regressions were performed to test the association between relative *w*AlbB and *w*Mel density and DENV-1 load at each dpi and viral titer.

## Results

### Primer evaluation for *Wolbachia* quantification

Melting curve analyses revealed single peaks for the *Ae. aegypti rps6* gene and *Wolbachia* *wsp* gene in both *w*Mel and *w*AlbB groups, suggesting that only one fragment is amplified by each primer pair in every reaction (Additional File 1: Figure S1). Primer efficiencies targeting the *rps6* and the *wsp* gene of *w*Mel and *w*AlbB were within the optimal range, with 103%, 92.3%, and 90.6% efficiency, respectively. Only *w*Mel-infected *Ae. aegypti* had amplification of the *wsp* gene when using primers designed for the *w*Mel strain. In the case of primers designed for the *w*AlbB strain, all *w*AlbB-infected mosquitoes had the *wsp* gene successfully amplified, with Ct ranging from 12 to 16; *Wolbachia*-free mosquitoes showed no amplification. *w*Mel-infected mosquitoes had spurious amplifications at Ct > 32 or no amplification.

### *Wolbachia* quantification in *Ae. aegypti* mosquitoes

**Fig. 1:**
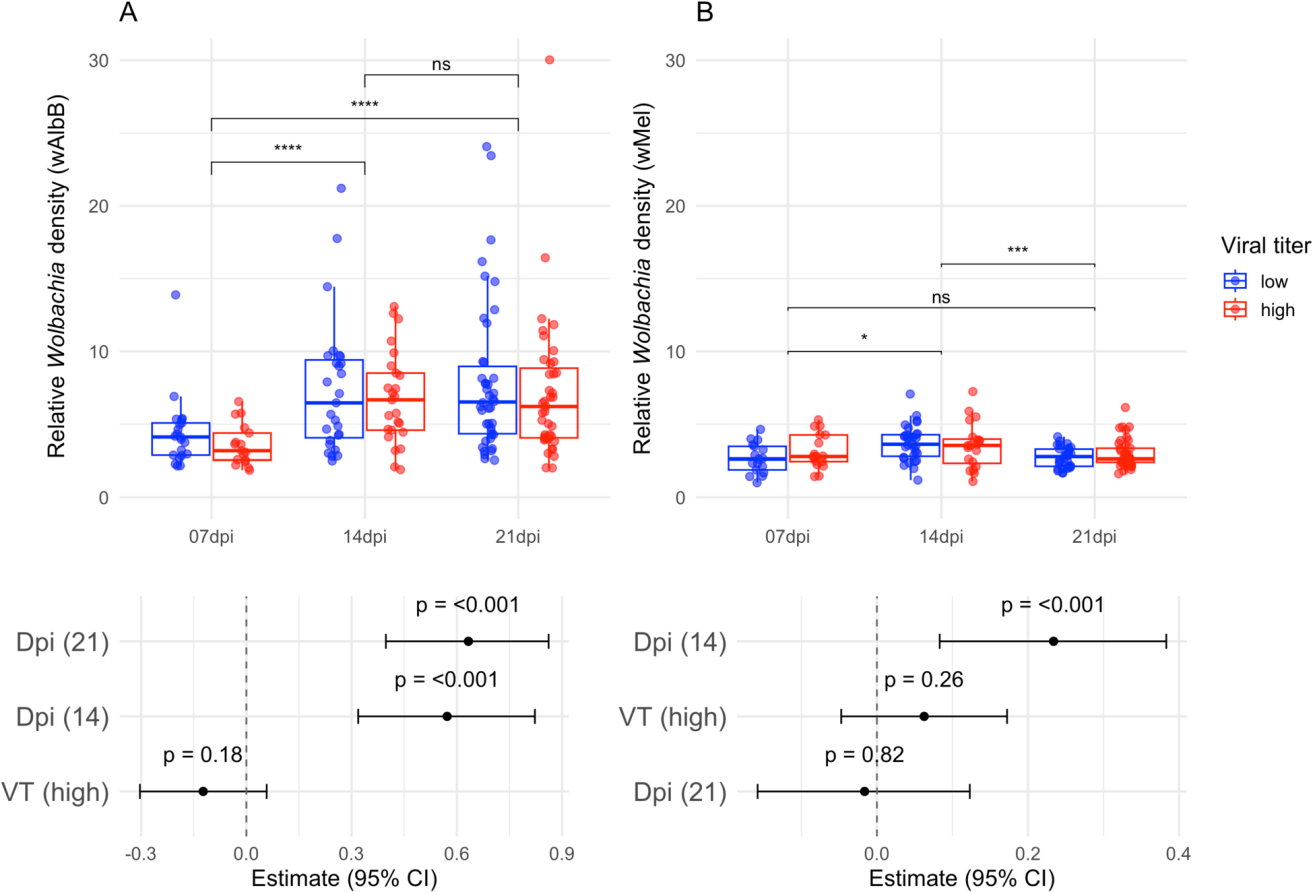
Relative *Wolbachia* density in *w*AlbB (**A**) and *w*Mel–*Ae. aegypti* (**B**) per days post infection (7, 14, and 21 dpi) and viral titer (VT; low and high). Each dot represents a tested mosquito. The panels below the plots show the results of GLMs with gamma distribution testing the association of relative *Wolbachia* density with dpi and viral titer. In this case, horizontal lines represent 95% confidence intervals for model regression coefficients (estimates). Relative *Wolbachia* density was compared over time post infection through Mann–Whitney tests. Significance level: ns non-significant ^*^ *P*-value < 0.05 ^***^ *P*-value < 0.001 and ^****^ *P*-value < 0.0001

A total of 493 *Ae. aegypti* females were exposed to two DENV-1 titers, of which 162 mosquitoes were wild, 174 were infected with *w*AlbB, and 157 were infected with *w*Mel. The relative *Wolbachia* density was quantified in each mosquito at 7, 14, and 21 days post infection (dpi) (Additional File 2: Table S1). The replicates were not statistically different from each other and thus analyzed as a single dataset (Additional File 2: Table S2). For mosquitoes infected with *w*AlbB*,* 98.3% (*n* = 171) of females tested positive for the bacteria (negative mosquitoes were then excluded from the analyses), while 100% of the *w*Mel *Ae. aegypti* were infected by this *Wolbachia* strain. For both *Wolbachia* strains, models without interactions between dpi and viral titer were retained for interpretation (Additional File 2: Table S3). No differences in *w*Mel or *w*AlbB densities were detected between mosquitoes subjected to different DENV-1 viral titers (Fig. 1, lower panels) and thus were pooled for statistical pairwise comparisons. Considering the *Ae. aegypti* group infected with *w*AlbB, relative *Wolbachia* density was higher at 14 and 21 dpi when compared with 7 dpi (7 dpi versus 14 dpi: *W* = 451, *P*-value < 0.01; 7 dpi versus 21 dpi: *W* = 666, *P*-value < 0.01), while no differences were detected between 14 and 21 dpi (14 dpi versus 21 dpi: *W* = 2110.5, *P*-value = 0.99; Fig. 1A). However, relative *w*Mel density was higher at 14 dpi than at 7 and 21 dpi (7 dpi versus 14 dpi: *W* = 598.5, *P*-value = 0.02; 14 dpi versus 21 dpi: *W* = 2491, *P*-value < 0.01) and similar at 7 and 21 dpi (7 dpi versus 21 dpi: *W* = 1218.5, *P*-value = 0.94; Fig. 1B).

**Fig. 2:**
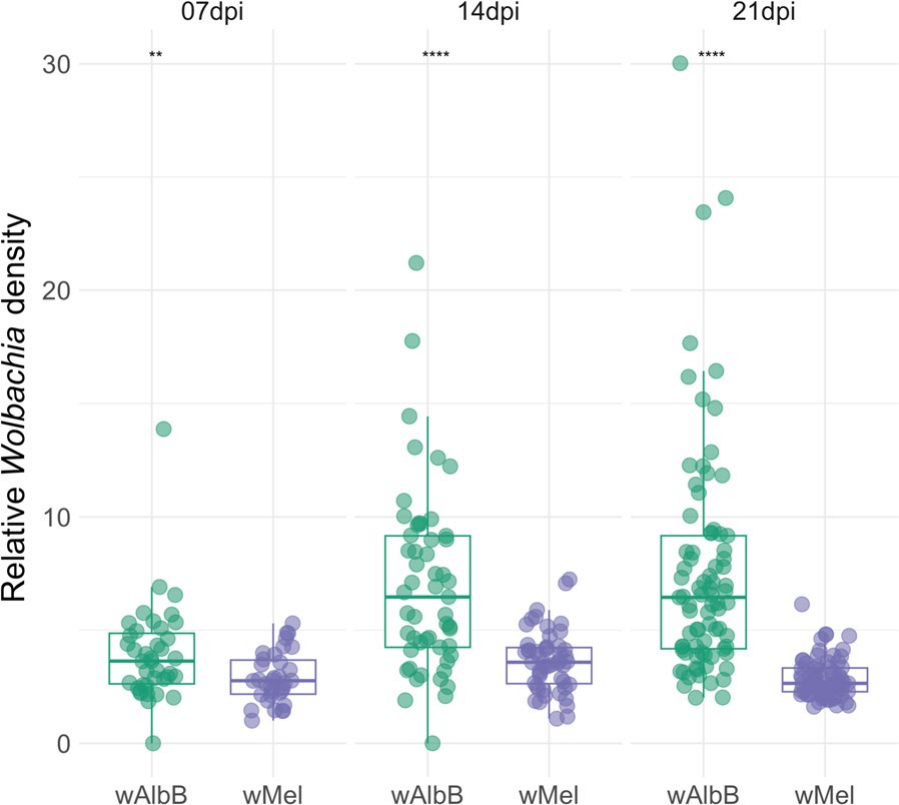
Relative *Wolbachia* density in *w*AlbB and *w*Mel–*Ae. aegypti* per days post infection (dpi). Each dot represents a tested mosquito, and the horizontal line represents the median relative *Wolbachia* density. Groups were compared at each dpi through Mann–Whitney tests. Significance level: ^**^ *P*-value < 0.01 and ^****^ *P*-value < 0.0001

Since the relative *Wolbachia* density varied over time but not between viral titers, groups were compared at each dpi. Our results indicated that the relative *w*AlbB density in *Ae. aegypti* was consistently higher than the relative *w*Mel density at all time points (7 dpi: *W* = 899, *P*-value = 0.01; 14 dpi: *W* = 2145, *P*-value < 0.01; 21 dpi: *W* = 5257, *P*-value < 0.01). Moreover, relative *Wolbachia* density was visually more heterogeneous between mosquitoes infected with *w*AlbB than for those infected with *w*Mel (Fig. 2).

### *Wolbachia* effect on *Ae. aegypti* body infection rates of DENV-1

In this section, we evaluated how *Wolbachia* presence affected DENV-1 infection rates in the body of *Ae. aegypti* on different days post infection (dpi). After being orally exposed to two different viral titers, 492 of 493 females (the RNA extraction from one *w*AlbB female failed) had their body tested for DENV infection via RT-qPCR at 7, 14, and 21 dpi, which included 162 wild, 173 *w*AlbB-, and 157 *w*Mel-infected mosquitoes. The two replicates were not statistically different from each other and thus analyzed as a single dataset (Additional File 2: Table S2). For the three time points, we reported the additive models without the interaction term between group and viral titer (Additional File 2: Table S4).

**Fig. 3:**
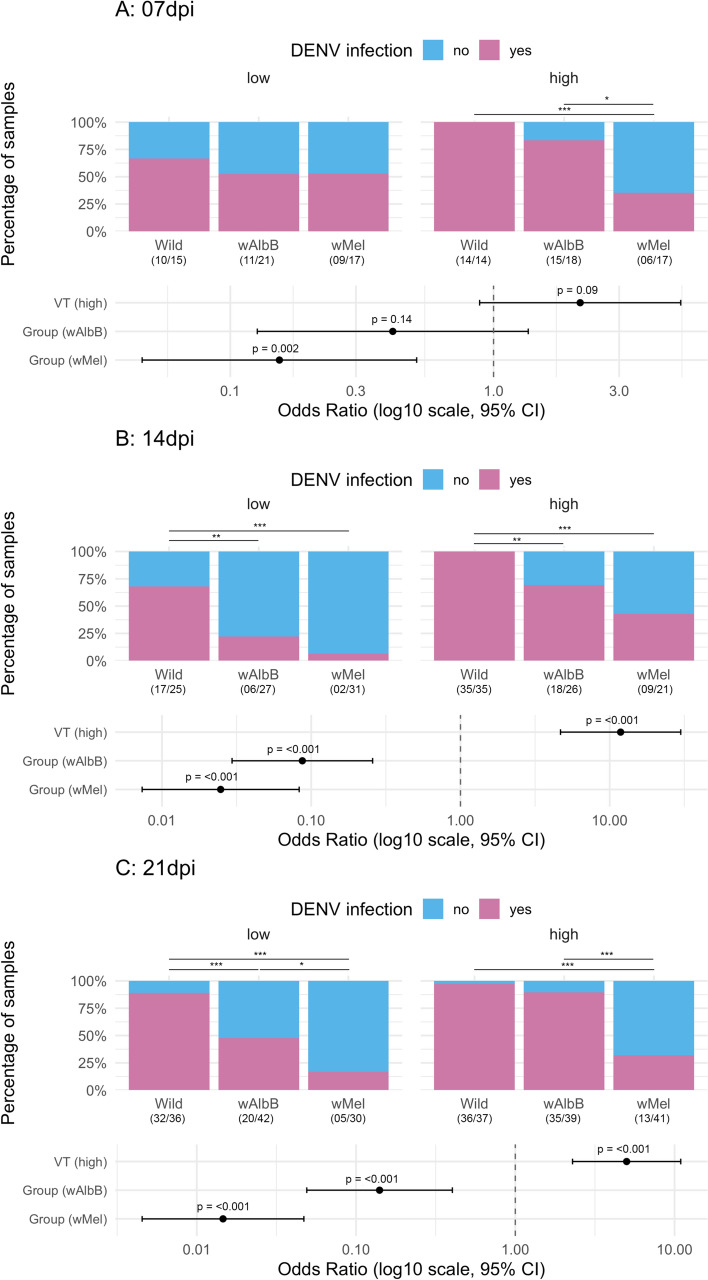
DENV-1 infection rates of mosquitoes’ bodies at 7 (**A**), 14 (**B**), and 21 (**C**) days post infection (dpi) per group (wild, *w*AlbB and *w*Mel) and viral titer (VT; low and high). Numbers in parenthesis indicate DENV-1 positive mosquitoes/total tested. Lower boxes show estimates (with 95% CI) and *P*-values from logistic regressions fitted for each dpi. The dashed line indicates the reference level (as odds ratio [OR] = 1): “wild” for group and “low” for viral titer. *P*-values are shown above the confidence intervals. Groups were compared at each dpi and viral titer through Fisher’s or chi-squared tests. Significance level: ^*^P-value < 0.05, ^**^P-value < 0.01, and ^***^P-value < 0.001. Nonsignificant differences are not shown

At 7 dpi, no differences were detected for DENV-1 infection rates in mosquito bodies between wild type and *w*AlbB-infected mosquitoes when considering both viral doses together, but a reduction in DENV-1 infection prevalence was observed for *w*Mel-infected *Ae. aegypti* in comparison with wild type individuals (Fig. 3A). The reduction in DENV-1 infection was more evident for the high viral titer, in which 100% of wild mosquitoes (*n* = 14) had detectable viral particles in their body, while only 35.3% of *w*Mel-infected mosquitoes (6 of 17) were infected with the virus. This scenario was supported in pairwise comparisons, of which the proportion of DENV-1-infected *Ae. aegypti* was significantly lower for the *w*Mel group in contrast to wild and *w*AlbB under the higher infection dose (*w*Mel versus wild: Fisher’s test < 0.01, *P*-value < 0.01; *w*Mel versus *w*AlbB: Fisher’s test = 0.12, *P*-value = 0.01; wild versus *w*AlbB: Fisher’s test = NA, *P*-value = 0.31).

A different scenario was observed at 14 dpi, as the DENV-1 infection rate in the mosquito’s body was lower for both *Wolbachia* groups when compared with the wild type group, regardless of the viral titer used (Fig. 3B). Pairwise comparisons supported this result (low titer: wild versus *w*AlbB: chi-squared = 9.25, *P*-value < 0.01; wild versus *w*Mel: Fisher’s test = 0.04; *P*-value < 0.01; high titer: wild versus *w*AlbB: Fisher’s test = NA, *P*-value < 0.01; wild versus *w*Mel: Fisher’s test = 0.00, *P*-value < 0.01), which also indicated similar DENV-1 infection rates in *w*AlbB- and *w*Mel-infected mosquitoes for both viral titers (low titer: *w*AlbB versus *w*Mel: Fisher’s test = 0.25, *P*-value = 0.18; high titer: *w*AlbB versus *w*Mel: chi-squared = 2.31, *P*-value = 0.18)*.*

At 21 dpi, both *Wolbachia* strains effectively reduced DENV-1 infection rate in mosquito’s body in contrast to the wild group when mosquitoes were challenged with the low viral titer (wild versus *w*AlbB: Fisher’s test = 8.55, *P*-value < 0.01; wild versus *w*Mel: Fisher’s test = 0.03, *P*-value < 0.01 Fig. 3C). When challenged with the high viral titer, however, only *w*Mel mosquitoes had a lower DENV-1 infection rate in their bodies (wild versus *w*Mel: Fisher’s test = 0.01, *P*-value < 0.01; wild versus *w*AlbB: Fisher’s test = 4.05, *P*-value = 0.43). All pairwise comparisons can be found in Additional File 2: Table S5.

DENV-1 infection rates in the body seemed to have distinct dynamics in wild, *w*Mel-, and *w*AlbB-infected mosquitoes. In wild type mosquitoes, infection rates were consistently higher under the high viral titer (97.3–100%), in contrast to the low titer (66.7–88.9%), and no significant variation was detected over time post infection (7, 14, and 21 dpi; Additional File 1: Figure S2A and Additional File 2: Table S4). For *w*AlbB-infected mosquitoes, the DENV-1 infection risk was significantly lower at 14 dpi compared with 7 dpi, and no differences were detected in DENV-1 infection rates over time after pairwise comparisons under high or low viral titer (Additional File 2: Table S4). Moreover, infection rates were always higher when exposed to the high viral titer (69.2–89.7%) than to the low viral titer (22.2–52.4%) (Additional File 1: Figure S2B and Additional File 2: Table S4). In *w*Mel-infected mosquitoes, DENV-1 infection rates declined at 14 and 21 dpi when compared with DENV-1 infection rates at 7 dpi, especially under low viral titer (dropping from 52.9% at 7 dpi to 6.5–16.7%), while the high titer led to a more stable but moderate infection rate (31.7–42.9%). Overall, the infection risk was consistently lower under low viral doses across all time points (Additional File 1: Figure S1B and Additional File 2: Table S4).

### *Wolbachia* effects on DENV-1 load in the body of *Ae. aegypti*

**Fig. 4:**
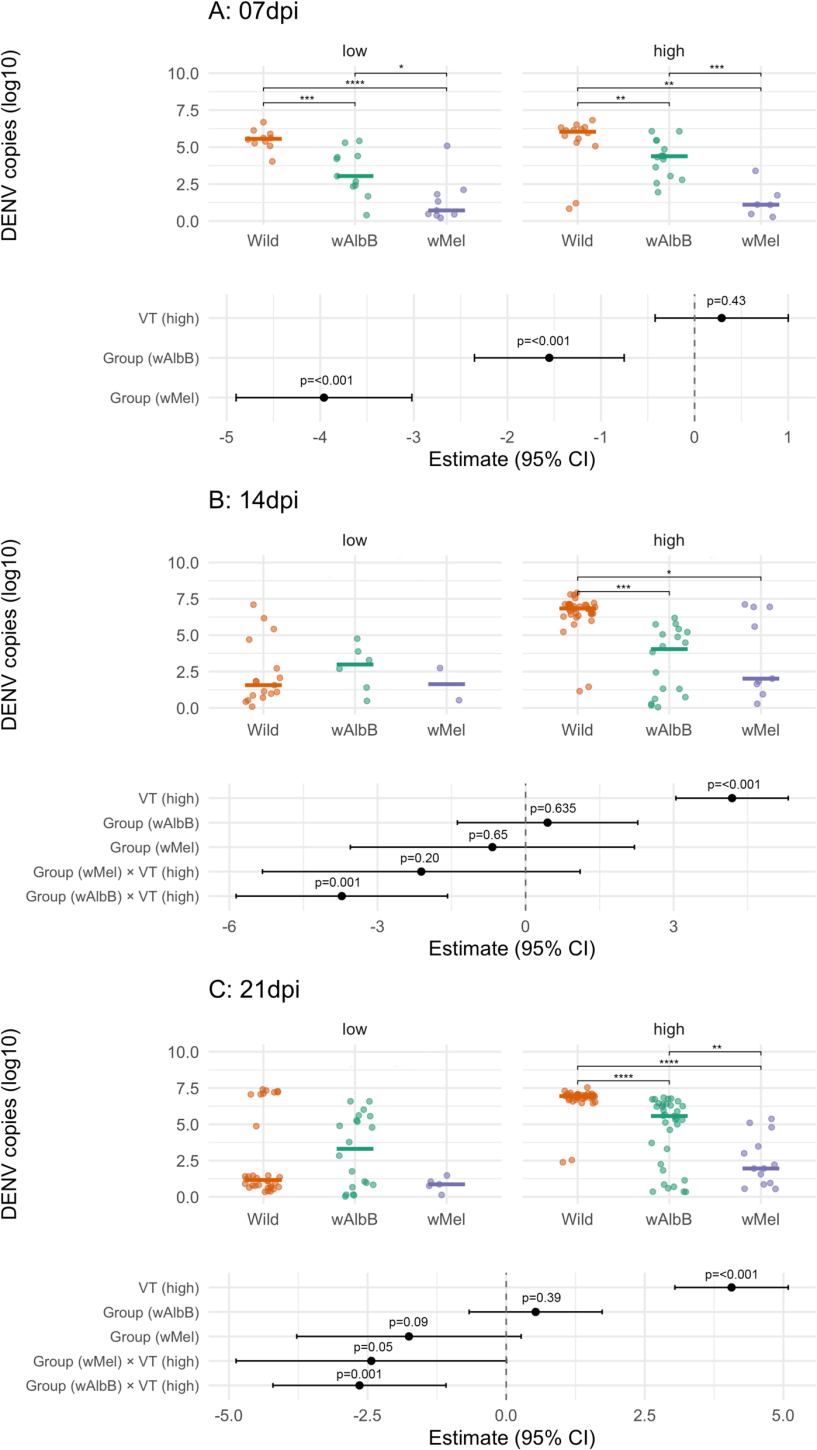
Upper plots show DENV copies (log_10_) in *Ae. aegypti* mosquito bodies at 7 (**A**), 14 (**B**), and 21 (**C**) days post infection (dpi), per group (wild, *w*AlbB, and *w*Mel), and viral titer (low and high). Each dot represents a tested mosquito, and the horizontal line represents the median DENV load value. Groups were compared at each dpi and viral titer through Mann–Whitney tests. Significance level: ^*^ *P* < 0.05; ^**^P < 0.01; ^***^P < 0.001; ^****^P < 0.0001. Lower plots show the results of linear regressions fitted for each dpi. The dashed line indicates the reference level (as zero): “wild” for group and “low” for viral titer. *P*-values are shown above the confidence intervals. Nonsignificant differences are not shown

In this section, we evaluated how *Wolbachia* infection affected the DENV-1 load in the body of *Ae. aegypti*. At 7 dpi, the linear model did include an interaction between viral titer and group (Additional File 2: Table S6). Overall, the DENV-1 load within groups did not change when comparing the different viral titers offered to mosquitoes (Fig. 4A). Regardless of the viral titers, both *Wolbachia*-infected groups had a reduction in viral copies relative to wild type mosquitoes, with a larger reduction in *Ae. aegypti* infected with *w*Mel than with *w*AlbB for both viral titers (Fig. 4A). Pairwise comparisons supported the reduction of viral loads in the body of mosquitoes infected with both *Wolbachia* strains when compared with the wild mosquitoes (low titer: wild versus *w*AlbB: *W* = 100, *P*-value < 0.01; wild versus *w*Mel: *W* = 88, *P*-value < 0.01; high titer: wild versus *w*AlbB: *W* = 166, *P*-value = 0.01; wild versus *w*Mel: *W* = 78, *P*-value < 0.01, Fig. 4A).

**Fig. 5:**
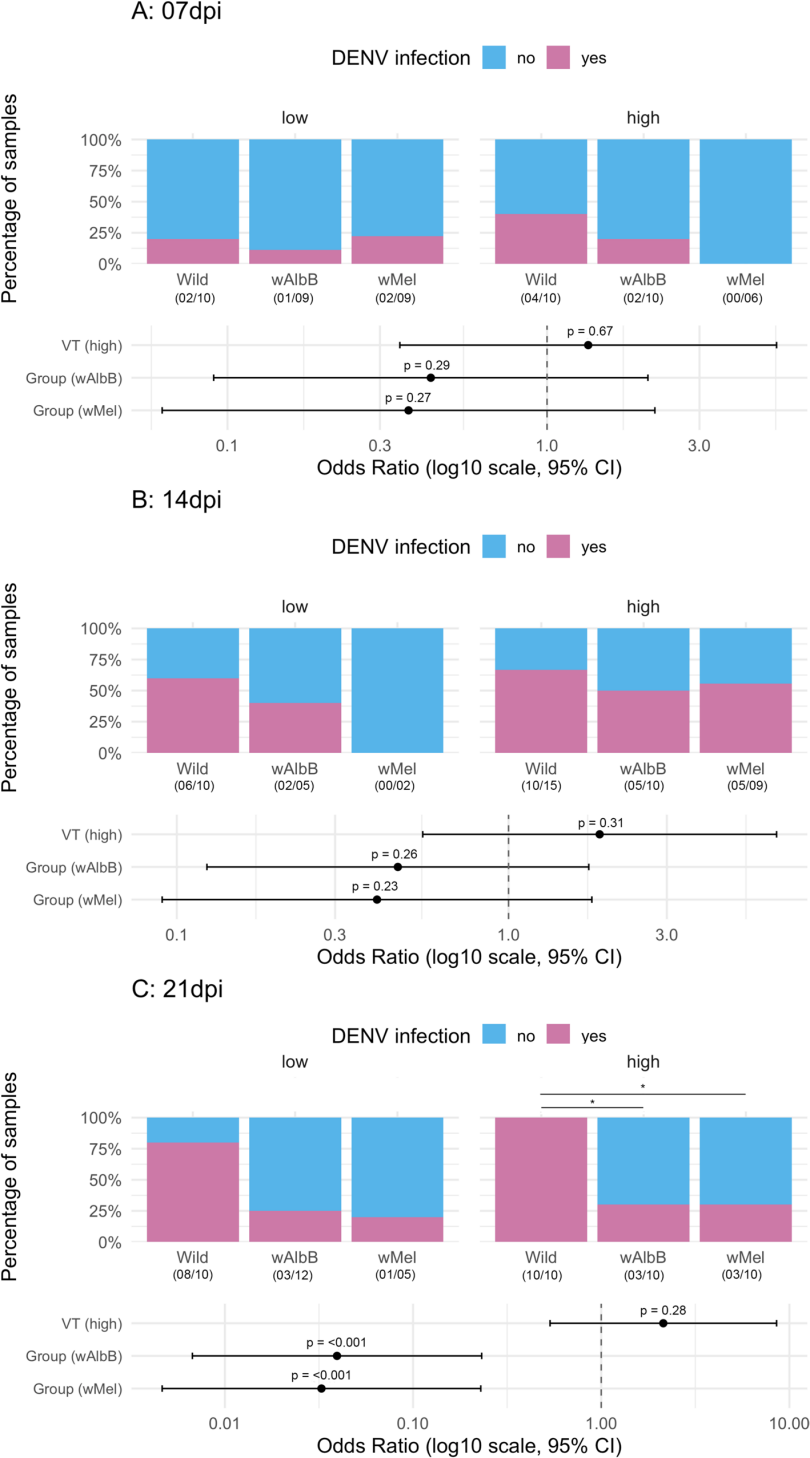
DENV-1 Transmission rates at 7 (**A**), 14 (**B**) and 21 (**C**) days post infection (dpi) per group (wild, *w*AlbB and *w*Mel) and viral titer (low and high). Lower boxes show estimates (with 95% CI) and *P*-values from logistic regressions fitted for each dpi. The dashed line indicates the reference level (as OR = 1): “wild” for group and “low” for viral titer. *P*-values are shown above the confidence intervals. Groups were compared at each dpi and viral titer through Fisher’s or chi-squared tests. Significance level: ^*^P-value < 0.05, ^**^P-value < 0.01, and ***P-value < 0.001. Nonsignificant differences are not shown

At 14 dpi, an interaction between explanatory variables (groups and viral titers) was retained in the model (Additional File 2: Table S6) and significant only for *w*AlbB-infected mosquitoes (Fig. 4B), which suggests that the influence of this bacteria strain on viral load was dose dependent. Significant differences in viral copies between *w*AlbB-mosquitoes and the wild type were observed only when mosquitoes were exposed to the high viral titer (wild versus *w*AlbB: *W* = 598, *P*-value < 0.01; Fig. 4B). Moreover, although the interaction between group (*w*Mel) and viral titer was not significant (Fig. 4B), pairwise comparisons showed a significant reduction in viral load under the high viral titer in comparison with wild *Ae. aegypti* (wild versus *w*Mel: *W* = 237, *P*-value = 0.03; Fig. 5B). A significant global effect of viral titer was revealed, indicating that mosquitoes challenged with the high viral titer also had higher DENV-1 loads in their bodies (Fig. 4B).

At 21 dpi, a similar result to that observed at 14 dpi was obtained, with the interaction between groups and viral titers being retained in the model and significant only for *w*AlbB mosquitoes (Additional File 2: Table S6; Fig. 4C); a significant difference between this group and the wild type occurred only under the high viral titer (wild versus *w*AlbB: W = 1168, *P*-value < 0.01) and not under the low titer (wild versus *w*AlbB: *W* = 309, *P*-value = 0.89; Fig. 4C). Though the interaction term was not statistically significant for the *w*Mel-infected *Ae. aegypti*, fewer viral copies were significantly detected under the high viral titer for this group in comparison with the wild type (wild versus *w*Mel: *W* = 346, *P*-value = 0.01; Fig. 4C). Overall, mosquitoes challenged with the high viral titer exhibited more DENV-1 copies in their bodies (Fig. 4B). Detailed model comparison and pairwise Mann–Whitney tests are presented in Additional File 2: Table S6. Linear regressions showed no correlation between the relative density of *w*AlbB or *w*Mel and DENV-1 load at any dpi (Additional File 2: Table S7).

We also explored how DENV-1 viral load in mosquito bodies changes over time by viral titer across groups. In wild type mosquitoes, a significant interaction between dpi and viral titer was observed, with viral loads decreasing after 14 and 21 dpi in contrast to 7 dpi under low titer, while the opposite trend was seen over time under the high titer exposure (Additional File 1: Figure S3A, Additional File 2: Table S8). For *w*AlbB mosquitoes, viral load was stable over time and consistently higher when challenged with the high DENV-1 titer (Additional File 1: Figure S3B). In *w*Mel mosquitoes, no significant differences were observed over time or between viral titers (Additional File 1: Figure S3C, Additional File 2: Table S8). These results suggest that infection dynamics differ between mosquito groups and are more influenced by viral dose in wild and *w*AlbB groups than in *w*Mel (Additional File 1: Figure S3, Additional File 2: Table S8).

### *Wolbachia* effects on transmission rates

Here, we evaluated how *Wolbachia* infection affected DENV-1 transmission rates (i.e., viral detection in saliva-microinjected mosquitoes) of *Ae. aegypti* at different dpi. After being exposed to two different viral titers, a total of 162 females had their saliva tested for DENV-1 infection via microinjection in female mosquitoes followed by RT-qPCR at 7, 14, and 21 dpi. These included 65 wild, 56 *w*AlbB-, and 41 *w*Mel-infected mosquitoes. There was no significant effect of adding the interaction term between group and viral titer in logistic models for any time point (Additional File 2: Table S8), indicating that differences in DENV-1 transmission rates between mosquito groups occurred regardless of the viral dose offered.

At 7 and 14 dpi, no effects of viral titer or difference in DENV-1 transmission rate between groups were observed (Fig. 5A and B). Pairwise comparisons did not show any variation in transmission rates based on *Wolbachia* presence in *Ae. aegypti* (Additional File 2: Table S8). However, at 21 dpi, there was a significant reduction in DENV-1 transmission probability by both *w*AlbB- and *w*Mel-infected *Ae. aegypti* in contrast to the wild type, whereas no effect of viral titer was observed (Fig. 5C). The pairwise comparisons corroborated this variation in transmission rates between groups but only under the high viral titer (low titer: wild versus *w*AlbB: Fisher’s test = 10.40, *P*-value = 0.18; wild versus *w*Mel: Fisher’s test = 0.08, *P*-value = 0.40; high titer: wild versus *w*AlbB: Fisher’s test = 0, *P*-value = 0.03; wild versus *w*Mel: Fisher’s test = 0, *P*-value = 0.03). The results of the other pairwise comparisons are presented in Additional File 2: Table S8.

We also investigated how the DENV-1 transmission rate varied over time for each mosquito group and viral titer offered during oral infection. Transmission rates of wild mosquitoes exhibited a distinct behavior in contrast to *Wolbachia*-infected *Ae. aegypti*. Even though there was a trend of increasing the DENV-1 transmission rate over time post infection for the wild type (Additional File 1: Figure S4A), pairwise comparisons were nonsignificant (Additional File 2: Table S9). There was no effect of dpi on DENV-1 transmission rate of *w*AlbB and *w*Mel-infected mosquitoes (Additional File 1: Figure S4B and C, Additional File 2: Table S9). Neither group showed any influence of viral titer on transmission rates (Additional File 1: Figure S4, Additional File 2: Table S10).

## Discussion

The deployment of *Wolbachia*-infected *Ae. aegypti* mosquitoes as a strategy to reduce DENV transmission has been implemented in several countries with reductions in case notifications following *Wolbachia* releases [22–24, 36]. To date, two *Wolbachia* strains have been successfully used in *Ae. aegypti* population replacement: *w*Mel and *w*AlbB [26, 37]. The success of these interventions is influenced by multiple factors, including the genetic compatibility between released and local mosquito populations, the specific *Wolbachia* strain used, host–microbe interactions, and environmental conditions, such as temperature and humidity [30, 38–42]. Among the key questions that emerge is whether the pathogen-blocking phenotype in *Ae. aegypti* is specific to the virus serotype/genotype and *Wolbachia* strain [43–45]. In this context, we evaluated the vector competence of *Ae. aegypti* mosquitoes with a Brazilian genetic background for DENV-1, in the presence of either the *w*Mel or *w*AlbB strain. Our findings indicate that both *Wolbachia* strains conferred a reduction in DENV-1 infection and transmission rates. However, mosquitoes infected with *w*Mel exhibited a greater degree of viral inhibition compared with those carrying *w*AlbB, indicating strain-specific differences in antiviral protection.

A central attribute underpinning the use of *Wolbachia* for arboviral control is its pathogen-blocking phenotype, whose efficacy is correlated with the intracellular bacterial density within *Ae. aegypti* hosts [46–48]. Although *Wolbachia* density does not appear to be negatively correlated with viral infection [49, 50], Corrêa-Antônio et al. [27] observed that a population of *Ae. aegypti* from Rio de Janeiro with high densities of *w*Mel exhibited lower DENV-1 infection rates in their bodies when compared with other mosquito populations with low densities of the bacterium. This finding suggests the existence of a density threshold of the bacterium required to achieve more effective arbovirus blocking [27]. In the present study, there was also no correlation between relative *w*Mel or *w*AlbB density and DENV-1 load, but *w*AlbB conferred overall lower inhibition of DENV-1 infection and transmission in *Ae. aegypti* mosquitoes with a Brazilian genetic background, despite exhibiting significantly higher densities compared with *w*Mel across all evaluated time points (7, 14, and 21 dpi; Fig. 2). These results indicate that the blocking phenotype depends on the bacterial strain rather than solely on its ability to invade and colonize mosquito tissues in high densities.

Another important factor influencing DENV-1 infection, and consequently the *Wolbachia*-mediated pathogen-blocking phenotype, is the viral titer received, as a higher inoculum facilitates systemic viral dissemination within the mosquito [51]. Indeed, 77.6% (59/76) of wild mosquitoes were infected with DENV-1 when exposed to a low viral titer (10^3^–10^4^ FFU/mL), while 98.8% (85/86) were infected when challenged with a high viral titer (10^4^–10^5^ FFU/mL) (Fig. 3). Similar infection dynamics were observed in *Wolbachia-*carrying mosquitoes, of which 20.5–41.1% had their bodies infected with DENV-1 when exposed to a low titer, while 35.4–81.9% were infected when offered the high viral titer (Fig. 3, Additional File 1: Figure S2). However, DENV-1 infection rates were different between the mosquitoes infected with distinct bacterial strains. In *w*Mel-infected mosquitoes, DENV-1 infection rates in mosquitoes’ bodies decreased significantly over time, particularly under the low-titer condition: rates dropped from 52.9% at 7 dpi to 6.5–16.7% at 14 and 21 dpi. Conversely, DENV-1 high-titer exposure produced more sustained, but moderate infection rates, ranging from 31.7% to 42.9% over the same period. For *w*AlbB-infected mosquitoes, infection rates remained consistently high when challenged with the high viral dose, across all time points. When exposed to low viral titer, a pronounced reduction was observed from 7 to 14 dpi (52.4% to 22%), but the infection rates increased at 21 dpi (47.6%).

The viral titers administered to mosquitoes through oral feeding are comparable with those detected in the blood of patients with asymptomatic DENV [52]. Similar levels can also occur in some symptomatic individuals with low viremia [53]. Mosquitoes infected with *w*AlbB maintained 10^3^–10^5^ DENV-1 copies in their bodies and their *w*Mel-infected counterparts exhibited a more stringent limit of 10^1^–10^2^ copies in all experiments, regardless of the different time points and the viral titers provided (Additional File 1: Figure S3). Wild mosquitoes when exposed to the low DENV-1 titer had a high viral levels at 7 dpi (10^5^ viral copies), but low levels at 14 and 21 dpi (~ 10^1^ viral copies), and thus *Wolbachia* had a protective effect only in the first time point analyzed, but when exposed to the high viral titer, DENV-1 levels in wild mosquitoes rose from 10^6^ at 7 dpi to 10^7^ at 14 and 21 dpi. In this case, the protective effect of *Wolbachia* was evident at all time points (Fig. 4). The fact that the viral loads in mosquitoes’ bodies infected with the *w*Mel and *w*AlbB strains remained relatively constant over time, regardless of the viral titer they were exposed to, suggests that *Wolbachia* may have a maximum pathogen-blocking capacity beyond which additional virus suppression cannot be achieved. The molecular pathway behind this phenotype warrants further investigation, since it directly determines the extent to which *Wolbachia* can limit viral amplification within mosquito populations, ultimately shaping the potential epidemiological impact of this intervention.

More definitive insights into *Wolbachia*-mediated dengue interference are obtained by analyzing viral presence in mosquito saliva, which serves as a proxy for transmission potential and hence the efficacy of the endosymbiont as a public health tool. No significant differences in DENV-1 transmission rates were detected between *Wolbachia* infected and uninfected groups at 7 dpi, when a low percentage of mosquitoes were infected (20–40% in wild and 0–22% in *Wolbachia* groups), or at 14 dpi, when 50–67% of wild and *Wolbachia*-infected mosquitoes exposed to DENV-1 high titer had infective saliva (Fig. 5). Although the low number of samples infected with DENV-1 with low titer at 14 dpi may weaken statistical comparisons, no significant differences were observed between the transmission rate of DENV-1 in wild mosquitoes (6/10 at 14 dpi) and their *Wolbachia*-infected counterparts (0/2 for *w*Mel and 2/5 for *w*AlbB at 14 dpi under the low viral titer, Fig. 5). This limitation of our study was due to the low number of *Wolbachia*-infected samples positive for DENV-1 in their bodies at these time points (Fig. 3). A significant difference between the groups was observed only at 21 dpi after exposure to the high titer, when both *w*Mel- and *w*AlbB-infected mosquitoes exhibited reduced transmission rates relative to wild counterparts (Fig. 5). This possible delayed viral blocking effect of *Wolbachia* raises concerns and warrants further investigation, since the extrinsic incubation period (EIP) of DENV is estimated to be 2–15 days at 30 °C [54]. In other words, *Ae. aegypti* females may already be capable of transmitting the virus before *Wolbachia*-mediated blocking becomes effective.

Despite numerous studies on the vector competence of *Ae. aegypti* for DENV when infected with *Wolbachia*, very few have specifically analyzed the DENV-1 serotype (as summarized in [27]). For instance, *Ae. aegypti* with Vietnam genetic background carrying the *w*Mel strain showed an approximately 50% reduction in DENV-1-infective saliva at 14 dpi [50, 52]. Similarly, Flores et al. [18] observed a small but significant reduction in the odds of DENV transmission potential in the saliva of Australian *Ae. aegypti* infected with either *w*Mel or *w*AlbB at 14 dpi (OR = 0.01–0.04, *P* < 0.001). These divergent results may be related to differences in virus genotypes and/or mosquito genetic backgrounds, and future work is necessary to assess their impacts on the effectiveness of the *Wolbachia* strategy across different geographical areas. Regardless of these variations, it is worth noting that the incomplete viral blockade could impose selective pressure on dengue populations, potentially favoring variants capable of efficient replication. Indeed, mutations in the DENV-1 envelope protein, such as E203K, can increase in frequency after repeated passages in *w*Mel-infected *Ae. aegypti* cells, suggesting that *Wolbachia* can influence viral evolution [55]. It remains unclear whether these variants can establish and spread in natural mosquito populations and thus genomic surveillance in areas with *Wolbachia* deployments is crucial [42].

Prior studies have demonstrated that the combination of host genotype and *Wolbachia* strain significantly influence the strength of viral blocking. For example, in *Ae. aegypti* with the Australian genetic background, *w*Mel and *w*AlbB produced comparable DENV-2 viral loads in whole-body samples [56]. Nonetheless, *w*AlbB-infected *Ae. aegypti* displayed a somewhat higher infection prevalence in abdomens and a lower prevalence in salivary glands in comparison with *w*Mel-infected specimens [57]. These findings are consistent with our data, suggesting that, while *w*AlbB may provide a weaker blocking of DENV infection at the midgut level compared with *w*Mel, it appears to confer a similar degree of viral inhibition at distal anatomical barriers, such as the salivary glands. In addition, comparative studies across all four DENV serotypes have shown that DENV-1 is more difficult to suppress than DENV-2, which could partially explain our results, especially the high DENV-1 infection rates observed here in the bodies of *w*AlbB-infected individuals [18].

Other factors can influence the success of implementing *Wolbachia* as a strategy for controlling arbovirus transmission. Environmental temperature is one of the most critical components that regulate the interaction between mosquito hosts, *Wolbachia* density, and viral blocking. In *Ae. aegypti*, *w*Mel is susceptible to thermal stress, with high larval rearing temperatures leading to reduced bacterial density and diminished expression of cytoplasmic incompatibility [58–60]. In contrast, *w*AlbB exhibits a markedly greater resilience to elevated rearing temperatures, maintaining density and cytoplasmic incompatibility expression under conditions that compromise *w*Mel performance [59, 60]. Moreover, *w*AlbB effectively blocked DENV-2 dissemination under thermal stress, whereas *w*Mel exhibited a significant reduction in antiviral activity [61]. Given that *w*AlbB may exhibit comparable DENV blocking to *w*Mel for some serotypes [15], its enhanced thermal tolerance highlights its epidemiological relevance for tropical endemic regions, where dengue transmission is intense and seasonal, with case incidence peaking during warmer months.

In Rio de Janeiro, *Wolbachia* releases occurred from 2015 to 2019, after Australian *Ae. aegypti* mosquitoes infected with *w*Mel had been backcrossed with the local mosquito population [30]. A total of 20 months following the cessation of *Wolbachia* deployments, *Wolbachia* frequency remained < 20% in field mosquitoes [32, 62]. In addition, approximately 25% of *w*Mel negative *Ae. aegypti* had the Australian mitochondrial haplotype, indicating the loss of the bacteria or a failure in maternal transmission [62]. Since thermal stress could affect *Wolbachia* density and its transmission to female offspring, *w*AlbB may represent a more suitable candidate for population replacement strategies in thermally stressful environments than *w*Mel, depending on the predominant serotype. The continuous monitoring of *Wolbachia* frequency and density dynamics in *Ae. aegypti* populations, particularly under field conditions and after the cessation of mosquito releases, are essential for evaluating the long-term effectiveness of arbovirus suppression programs and understanding fluctuations in vector competence following partial or full introgression of the symbiont.

## Conclusions

This study found that *w*Mel and *w*AlbB *Wolbachia* strains both reduce DENV-1 infection and transmission in *Ae. aegypti* with a Brazilian genetic background but with different efficacy. Despite lower bacterial density in mosquitoes’ bodies, *w*Mel conferred stronger viral blockade, while *w*AlbB reached higher densities but overall weaker blocking. These findings highlight that the pathogen suppression is *Wolbachia* strain-dependent rather than density-driven and may reach an upper limit beyond which additional viral reduction is not achieved. The incomplete blockage raises concerns about selective pressures on DENV-1 and the potential emergence of adaptive variants, emphasizing the need for genomic monitoring in *Wolbachia*-release areas. Environmental factors, particularly temperature, also play a decisive role, with *w*AlbB showing greater thermal resilience than *w*Mel [59], suggesting its suitability for tropical regions. Long-term surveillance of *Wolbachia* frequency and density in the field will be critical to assess the durability and epidemiological impact of this strategy.

## Supplementary Information

Supplementary Material 1.

Supplementary Material 2.

## Data Availability

All data generated and analyzed during this study are included in this published article and its supplementary information files.
