# Supplementary material for: Wolbachia wMel and wAlbB strains differentially impact the vector competence of Aedes aegypti with a Brazilian genetic background for DENV-1 virus
Source: Parasit Vectors. 2026 Mar 5;19:149. doi: 10.1186/s13071-026-07292-6 (PMC13064027; doi:10.1186/s13071-026-07292-6)
Supplement: Supplementary file 1 — Supplementary Material 1. [file 13071_2026_7292_MOESM1_ESM.docx]

***Wolbachia* *w*Mel and *w*AlbB strains differentially impacts the vector competence of *Aedes aegypti* with Brazilian genetic background for DENV-1 virus**

**Carolina Boucinha Martins^1,#^, Mariana Rocha David^1,#^, Dinair Couto-Lima^1^, Jessica Corrêa-Antônio^1^, Rayane Teles-de-Freitas^1^, Manuella Mello-Barbosa^1^, Renke Lühken^2^, Ary Hoffmann^3^, Rafael Maciel-de-Freitas^1,2,*^, Márcio Galvão Pavan^1,*^**

^1^ Laboratório de Mosquitos Transmissores de Hematozoários, Instituto Oswaldo Cruz, Fiocruz, Rio de Janeiro, Brazil.

^2^ Department of Entomology and Arbovirology, Bernhard Nocht Institute for Tropical Medicine, Hamburg, Germany.

^3^ Pest and Environmental Adaptation Research Group, School of BioSciences, Bio21 Institute, The University of Melbourne, Melbourne, Australia.

**^#^ Equal contribution**

*** Correspondence**: [mgpavan@ioc.fiocruz.br](mailto:mgpavan@ioc.fiocruz.br), [macieldefreitas@bnitm.de](mailto:macieldefreitas@bnitm.de)

**Additional File 1: Supplementary Figures**


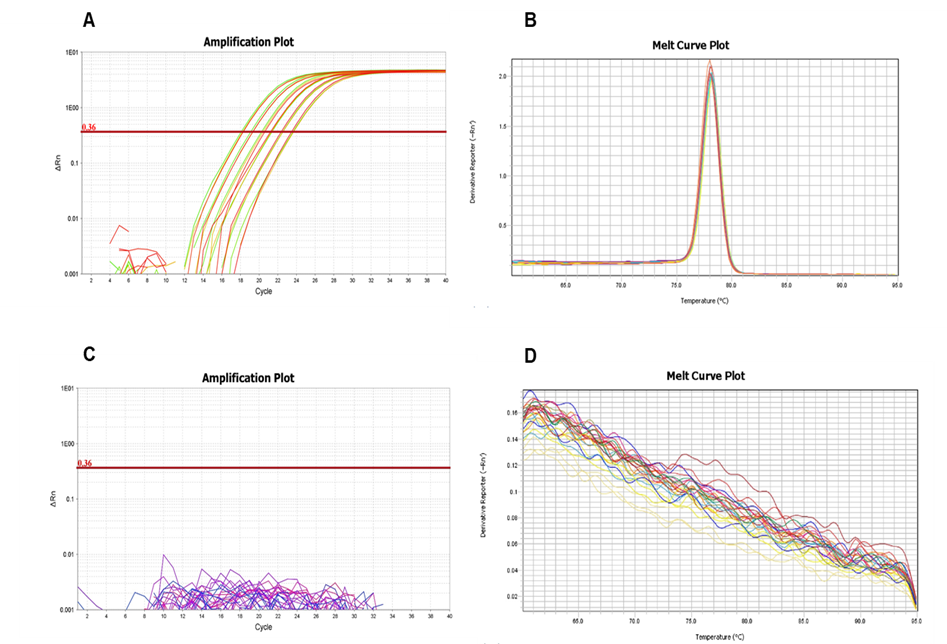


**Figure S1. Oligonucleotides for the *wsp* gene of *w*AlbB.** (A) Amplification curve for the *wsp* gene of *Wolbachia* *w*AlbB strain, using DNA from *Ae. aegypti* females infected with *w*AlbB. (B) Melting curve for the *wsp* gene of *Wolbachia* *w*AlbB, using DNA from *Ae. aegypti* females infected with *w*AlbB. (C) Amplification curve for the *wsp* gene of *w*AlbB, using DNA from *Wolbachia*-free *Ae. aegypti* females (*PAEA* strain). (D) Melting curve for the *wsp* gene of *w*AlbB, using DNA from *Wolbachia*-free *Ae. aegypti* females (*PAEA* strain). Amplification curves and melting curves for the other oligonucleotides used in this study are provided elsewhere [32], and single peaks were consistently observed in melting curves of all other primer sets.


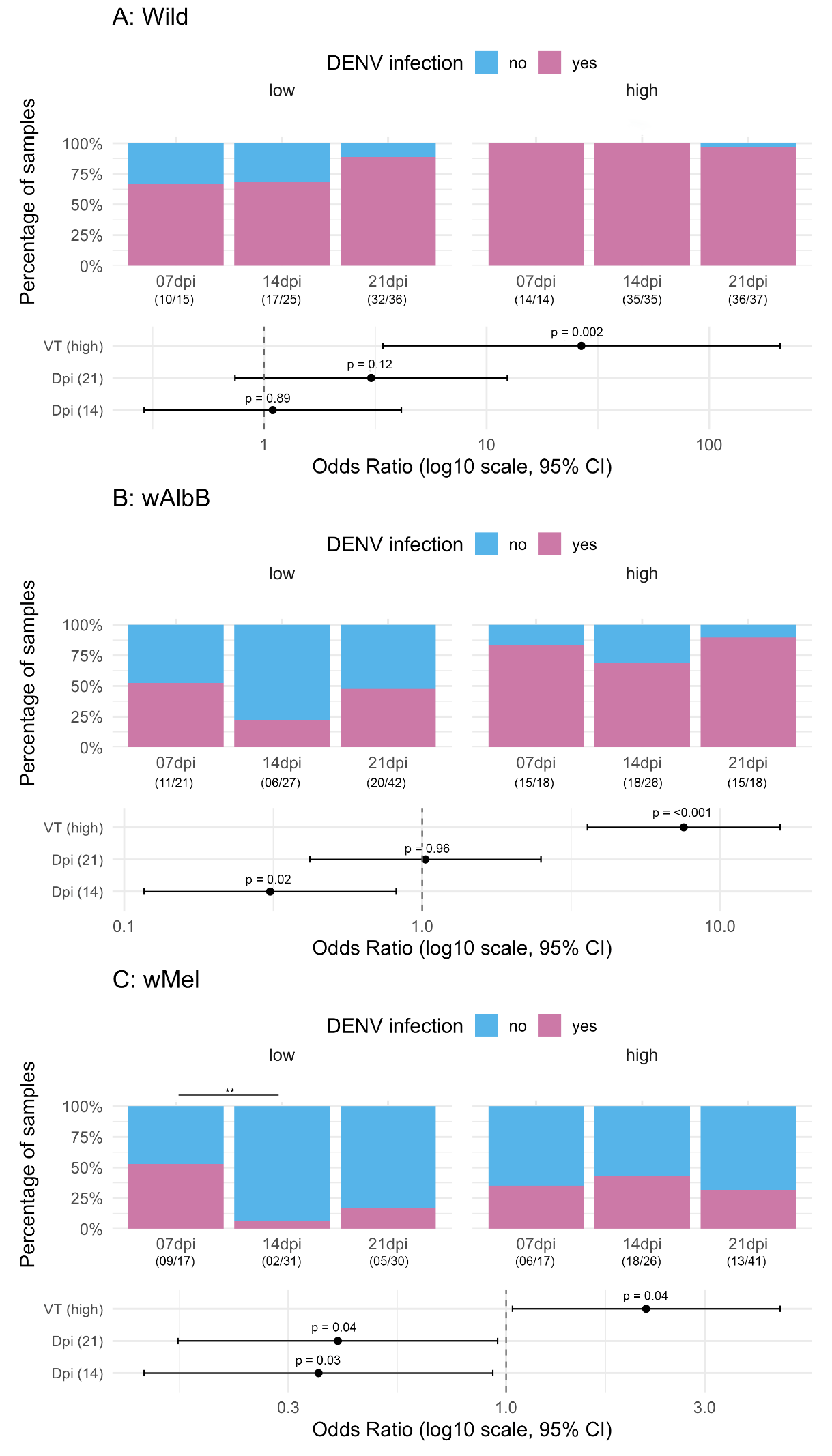


**Figure S2.** **DENV-1 infection rates of mosquito’s bodies for wild (A), *w*AlbB- (B) and *w*Mel-infected *Ae. aegypti* (C) per day post infection (dpi) and viral titer (low and high).** Numbers in parenthesis indicate DENV-1 positive mosquitos/total tested. Timepoints (dpi) were compared for each group and viral titer through Fisher’s or Chi-Squared tests. Significance level: * p-value < 0.05, ** p-value < 0.01 and *** p-value < 0.001. Non-significant differences are not shown. The panels below the plots show the results of logistic regressions testing the association of body infection rate with dpi and viral titer. In this case, horizontal lines represent 95% confidence intervals for model coefficients (estimates) with p-values. The interaction term did not improve model fit for any group (Table S3). The dashed line indicates the reference level (as OR = 1): “07 dpi” for dpi and “low” for viral titer. P-values are shown above the confidence intervals.


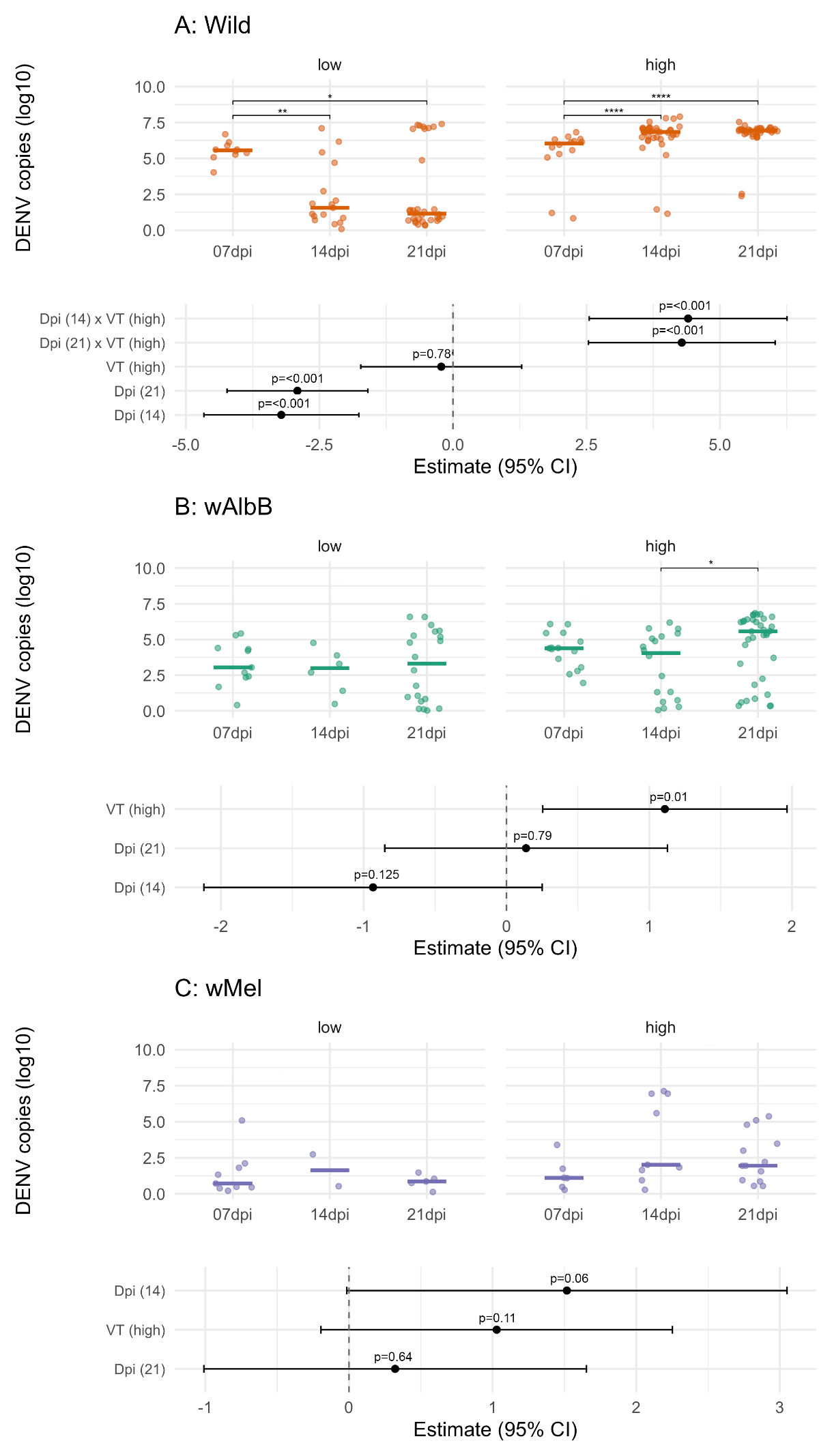


**Figure S3. Upper plots show DENV copies (log10) in *Ae. aegypti* mosquito bodies for wild (A), *w*AlbB (B) and *w*Mel-*Ae. aegypti* (C) per day post infection (dpi) and viral titer (VT).** Each dot represents a tested mosquito and the horizontal line represents the median DENV load value. Days post infection (dpi) were compared for each group and viral titer through Mann-Whitney tests. Significance level: * p < 0.05; ** p < 0.01; *** p < 0.001; **** p < 0.0001. Non-significant differences are not shown. The panels below the plots show the results of GLMs testing the association of body infection rate with dpi and viral titer. In this case, horizontal lines represent 95% confidence intervals for model coefficients (estimates) with p-values. The linear model for the wild group was the only case in which the interaction term was retained (Table S5). The dashed line indicates the reference level (as zero): “07” for dpi and “low” for viral titer. P-values are shown above the confidence intervals.


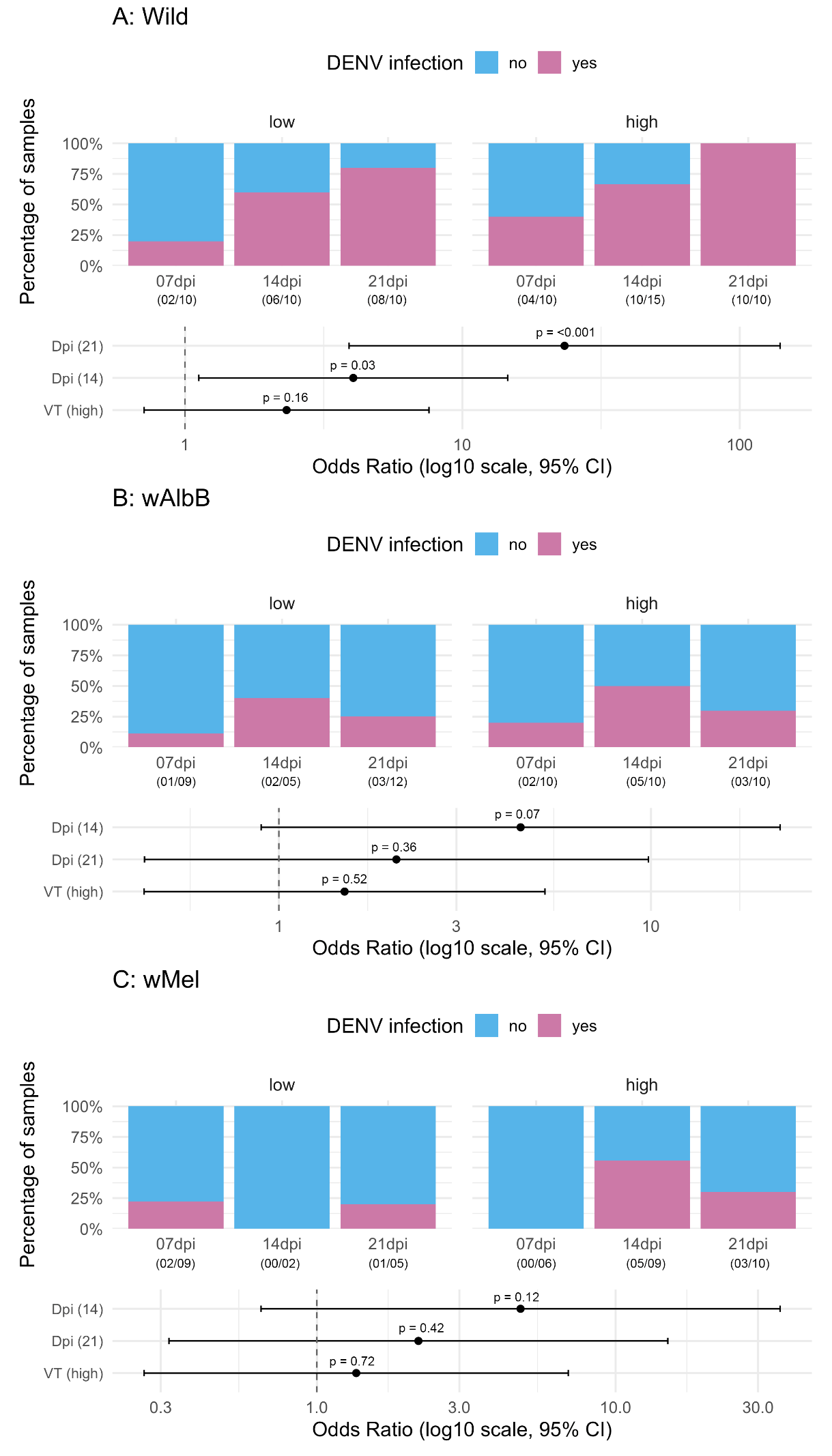


**Figure S4. DENV-1 transmission rates for wild (A), *w*AlbB (B) and *w*Mel-*Ae. aegypti* (C) per day post infection (dpi) and viral titer (low and high).** Timepoints (dpi) were compared for each group and viral titer through Fisher’s or Chi-Squared tests. Significance level: * p-value < 0.05, ** p-value < 0.01 and *** p-value < 0.001. Non-significant differences are not shown. The panels below the plots show the results of logistic regressions testing the association of transmission rate with dpi and viral titer. In this case, horizontal lines represent 95% confidence intervals for model coefficients (estimates) with p-values. The interaction term did not significantly improve model fit for any group (Table S7). The dashed line indicates the reference level (as OR = 1): “07 dpi” for dpi and “low” for viral titer. P-values are shown above the confidence intervals.
